# *In vivo* evaluation of the effects of simultaneous inhibition of GLUT-1 and HIF-1α by antisense oligodeoxynucleotides on the radiosensitivity of laryngeal carcinoma using micro ^18^F-FDG PET/CT

**DOI:** 10.18632/oncotarget.16671

**Published:** 2017-03-29

**Authors:** Li-Fang Shen, Xin Zhao, Shui-Hong Zhou, Zhong-Jie Lu, Kui Zhao, Jun Fan, Min-Li Zhou

**Affiliations:** ^1^ Department of Otolaryngology, The First Affiliated Hospital, College of Medicine, Zhejiang University, Hangzhou City, Zhejiang Province, China; ^2^ Center of PET/CT, The First Affiliated Hospital, College of Medicine, Zhejiang University, Hangzhou City, Zhejiang Province, China; ^3^ Department of Radiotherapy, The First Affiliated Hospital, College of Medicine, Zhejiang University, Hangzhou City, Zhejiang Province, China; ^4^ State Key Laboratory for Diagnosis and Treatment of Infectious Diseases, The First Affiliated Hospital, College of Medicine, Zhejiang University, Hangzhou City, Zhejiang Province, China

**Keywords:** hypoxia-inducible factor 1α, glucose transporter-1, antisense oligodeoxynucleotides, radiosensitivity, ^18^F-FDG micro PET/CT

## Abstract

**Purpose:**

Hypoxia-inducible factor 1α (HIF-1α) and glucose transporter-1 (GLUT-1) are two important hypoxic markers associated with the radioresistance of cancers including laryngeal carcinoma. We evaluated whether the simultaneous inhibition of GLUT-1 and HIF-1α expression improved the radiosensitivity of laryngeal carcinoma. We explored whether the expression of HIF-1α and GLUT-1 was correlated with 2′-deoxy-2’-[18F]fluoro-D-glucose (^18^F-FDG) uptake and whether ^18^F-FDG positron emission tomography-computed tomography (PET/CT) was appropriate for early evaluation of the response of laryngeal carcinoma to targeted treatment *in vivo*.

**Materials and Methods:**

To verify the above hypotheses, an *in vivo* model was applied by subcutaneously injecting Hep-2 (2 × 107/mL × 0.2 mL) and Tu212 cells (2 × 107/mL × 0.2 mL) into nude mice. The effects of HIF-1α antisense oligodeoxynucleotides (AS-ODNs) (100 μg) and GLUT-1 AS-ODNs (100 μg) on the radiosensitivity of laryngeal carcinoma were assessed by tumor volume and weight, microvessel density (MVD), apoptosis index (AI) and necrosis *in vivo* based on a full factorial (2^3^) design. ^18^F-FDG-PET/CT was taken before and after the treatment of xenografts. The relationships between HIF-1α and GLUT-1 expression and ^18^F-FDG uptake in xenografts were estimated and the value of ^18^F-FDG-PET/CT was assessed after treating the xenografts.

**Results:**

10 Gy X-ray irradiation decreased the weight of Hep-2 xenografts 8 and 12 days after treatment, and the weights of Tu212 xenografts 8 days after treatment. GLUT-1 AS-ODNs decreased the weight of Tu212 xenografts 12 days after treatment. There was a synergistic interaction among the three treatments (GLUT-1 AS-ODNs, HIF-1α AS-ODNs and 10Gy X-ray irradiation) in increasing apoptosis, decreasing MVD, and increasing necrosis in Hep-2 xenografts 8 days after treatment (*p* < 0.05) and in Tu212 xenografts 12 days after treatment (*p* < 0.001). Standardized uptake value (tumor/normal tissue)( SUVmaxT/N) did not show a statistically significant correlation with GLUT1 and HIF-1α expression and therapeutic effect (necrosis, apoptosis).

**Conclusions:**

Simultaneous inhibition of HIF-1α and GLUT-1 expression might increase the radiosensitivity of laryngeal carcinoma, decreasing MVD, and promoting apoptosis and necrosis. ^18^F-FDG-PET/CT wasn't useful in evaluating the therapeutic effect on laryngeal cancer in this animal study.

## INTRODUCTION

Although promising therapeutic strategies have been described, the poor overall survival rate of patients with laryngeal carcinoma remains unchanged [1]. One possible cause may be radioresistance of laryngeal carcinomas. The underlying mechanisms of radioresistance is still unclear and involves multiple factors including tumor cell proliferation, hypoxia and intrinsic radioresistance [2, 3].

Among them, hypoxia is an important issue [4]. HIF-1α is important factor induced during the adaptive response to hypoxia [5]. HIF-1α regulates multiple aspects of tumorigenesis, including proliferation, differentiation, angiogenesis, metabolism, metastasis, and responses to radiation therapy, making it a key regulator of malignant tumor phenotypes [6, 7]. HIF-1α has been associated with a poor prognosis in laryngeal carcinoma [8]. It has been reported that high expression of HIF and other endogenous hypoxia-related proteins was associated with radioresistance and a worse overall survival rate [5, 9]. Accordingly, HIF-1α has been suggested as a potential therapeutic target to improve radiosensitivity *in vitro* and *in vivo* [10, 11].

To our knowledge, there is only one report on targeting HIF-1α to enhance radiosensitivity in laryngeal cancer [12]. However, it was an *in vitro* study and the HIF-1α inhibitor used was not specific. Thus, the role of HIF-1α in laryngeal carcinoma radioresistance and whether inhibition of HIF-1α expression can improve radiosensitivity of laryngeal carcinoma require further evaluation.

At least one study has shown the limitations associated with inhibiting HIF-1α alone to improve radiosensitivity [13], thus, more effective strategies to enhance the radiosensitivity of laryngeal carcinoma need to be investigated. It may be useful to inhibit HIF-1 downstream target genes, including GLUT-1. GLUT-1 has been considered a possible intrinsic marker of hypoxia in malignant tumors, including laryngeal carcinoma [14, 15] Some studies have also demonstrated that increased GLUT-1 expression was associated with radioresistance [16, 17]. Our previous findings showed that GLUT-1 AS-ODNs inhibited glucose uptake and the proliferation of Hep-2 cells [18], and that GLUT-1 over-expression was associated with radioresistance in laryngeal cancer, furthermore, suppressing the expression of GLUT-1 may enhance the radioresistance of laryngeal carcinoma [19]. These results suggest that GLUT-1 expression is a marker of radioresistance in malignant tumors.

Although Amann et al proposed that combined inhibition of HIF-1α and GLUT-1 may be a novel therapeutic stategy in hepatocellular carcinoma [20], there is no report on the simultaneous inhibition of HIF-1α and GLUT-1 in laryngeal cancer. In this study, we assessed the effect of simultaneous inhibition of HIF-1α and GLUT-1 expression on radioresistance in laryngeal carcinomas *in vivo*. 2^3^ factorial design is adopted in this study concerning with the effects of formulation variables and their interactions on response variables to obtain the optimized formulation.

PET is a quantitative molecular imaging technique that allows noninvasive imaging *in vivo* and quantification of biological processes [21]. A microPET/CT scanner for animal studies has provided a novel technology for molecular imaging assays of metabolism and signal transduction [22].

The relationships between GLUT1, HIF1α expression and ^18^F-FDG uptake in head and neck squamous cell carcinoma(HNSCC) remain controversial. In our previous study, the expression of GLUT1 and HIF1α was significantly correlated with ^18^F-FDG uptake in patients with laryngeal carcinoma [23]. However, Mason et al reported that ^18^F-FDG uptake in HNSCC xenografts might not reflect the level of metabolic activity characteristic of HNSCC [24].

A few studies have investigated whether micro PET/CT is useful for determining radiosensitivity in nasopharyngeal carcinoma [25], and human glioblastoma [26] *in vivo*. In this study, we evaluated the radiosensitivity of laryngeal carcinoma using micro PET/CT and assessed the relationships between HIF1α, GLUT1-1 expression and 18F-FDG uptake *in vivo*.

## RESULTS

### General observations, volume, weight of the Hep-2 and Tu212 xenografts

The volumes of Hep-2 and Tu212 xenografts reached 100 mm^3^ at 9 days and 11 days after inoculation respectively. Mice in each group exhibited no obvious abnormalities in mental behavior, eating habits, defecation, weight or mortality during the experimental period.

**Figure 1:**
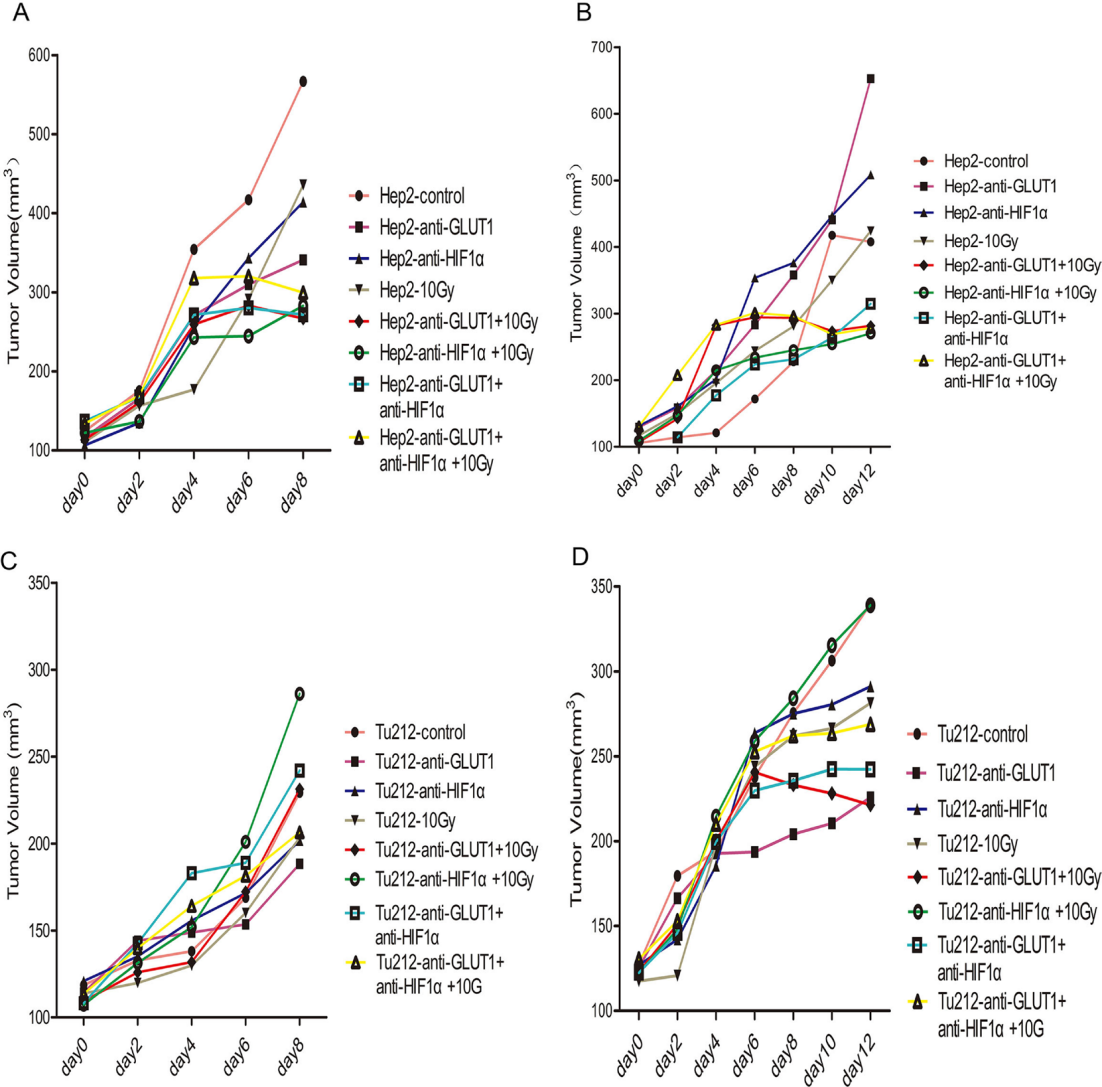
Tumor volumes were calculated every 2 days The volumes of Hep-2 xenografts 8 days after treatment (**A**), Hep-2 xenografts 12 days after treatment (**B**), and Tu212 xenografts 8 days after treatment (**C**) and Tu212 xenografts 12 days after treatment (**D**).

Mice were sacrificed 8 days after treatment initiation. The main effect of 10 Gy X-ray irradiation on tumor weight was 0.158 ± 0.03 g (*p* = 0.002) in Hep-2 xenografts, 0.05 ± 0.016 g (*p* = 0.038) in Tu212 xenografts, respectively (Table 1). At 12 days after treatment, the main effect of 10 Gy X-ray- irradiation on tumor weight was 0.208 ± 0.058 g in Hep-2 xenografts (*p* = 0.022), and the main effect of GLUT-1 AS-ODNs was 0.115 ± 0.025 g in Tu212 xenografts (*p* = 0.006) (Table 2). GLUT-1 AS-ODNs, HIF-1α AS-ODNs and 10Gy X-ray irradiation showed no interaction effects or main effects on the volumes of Hep-2 or Tu212 xenografts (*P* > 0.05) (Figure 1).

**Table 1:** Observed responses of xenografts 8 days after treatment in the 2^3^ factorial design with three independent parameters and their two levels (GLUT-1 AS-ODNs and HIF-1α AS-ODNs 100 μg or 0 μg, X-ray irradiation 10 Gy or 0 Gy)

G	A (μg)	B (μg)	C (Gy)	Tumor weight^x^ (g)		AI (%)^x^		MVD^x^		Necrosis ratex (%)	
				Hep-2	Tu212	Hep-2	Tu212	Hep-2	Tu212	Hep-2	Tu212
G1	100	100	10	0.36± 0.03	0.11 ± 0.01	47.71 ± 3.38	38.10 ± 0.41	3.67 ± 2.10	1.33 ± 0.66	51.67 ± 7.26	63.33 ± 3.33
G2	100	100	0	0.27 ± 0.08	0.13 ± 0.02	30.45 ± 2.51	31.66 ± 0.71	6.00 ± 1.10	6.33 ± 0.88	10.00 ± 2.88	23.33 ± 6.01
G3	0	100	10	0.57 ± 0.06	0.10 ±0.01	8.67 ± 1.35	9.70 ± 1.03	4.67 ± 0.88	4.33 ± 0.33	8.33 ± 1.66	23.33 ± 1.66
G4	100	0	10	0.41 ± 0.09	0.09 ± 0.01	14.04 ± 2.19	17.72 ± 1.41	11.33 ± 1.85	10.67 ± 1.33	10.00 ± 5.00	40.00 ± 2.88
G5	0	0	10	0.25 ± 0.03	0.11 ± 0.02	4.26 ± 0.53	5.68 ± 1.61	15.67 ± 1.76	12.00 ± 3.00	8.33 ± 1.66	35.00 ± 7.63
G6	0	100	0	0.21 ± 0.06	0.18 ± 0.06	6.07 ± 0.04	7.75 ± 1.05	13.00 ± 1.15	11.67 ± 1.76	5.00 ± 0.00	15.00 ± 5.00
G7	100	0	0	0.16 ± 0.01	0.13 ± 0.02	10.41 ± 0.51	14.85 ± 1.27	16.33 ± 2.18	18.33 ± 2.72	10.00 ± 2.88	5.00 ± 0.00
G8	0	0	0	0.33 ± 0.04	0.16 ± 0.04	2.24 ± 0.11	4.74 ± 1.28	34.00 ± 2.64	34.00 ± 3.05	1.66 ± 1.66	0.00 ± 0.00
a (*p*-value)				0.427	0.275	< 0.001	< 0.001	< 0.001	< 0.001	< 0.001	< 0.001
b (*p*-value)				0.09	0.711	< 0.001	< 0.001	< 0.001	< 0.001	< 0.001	0.591
c (*p*-value)				0.002	0.038	< 0.001	0.003	< 0.001	< 0.001	< 0.001	< 0.001
ab (*p*-value)				0.346	0.941	< 0.001	< 0.001	0.015	0.145	0.002	0.318
ac (*p*-value)				0.83	0.379	0.005	0.047	0.002	0.009	0.007	< 0.001
bc (*p*-value)				0.156	1.000	0.012	0.245	0.026	0.007	0.002	0.003
abc (*p*-value)				0.13	0.555	0.018	0.344	0.174	0.05	<0.001	0.004

G: group, A: GLUT-1 AS-ODNs, B: HIF-1α AS-ODNs, C: X-ray irradiation.

a: main effect of GLUT-1 AS-ONDs, b: main effect of HIF-1α AS-ODNs, c: main effect of X-ray irradiation, ab: interaction effect between GLUT-1 AS-ONDs and HIF-1α AS-ODNs, ac: interaction effect between GLUT-1 AS-ONDs and X-ray irradiation, bc: interaction effect between HIF-1α AS-ODNs and X-ray irradiation, abc: interaction effect between GLUT-1 AS-ONDs and HIF-1α AS-ODNs and X-ray irradiation.

^X^ Mean ± Std(standard deviation).

**Table 2:** Observed responses of xenografts 12 days after treatment in the 2^3^ factorial design with three independent parameters and their two levels (GLUT-1 AS-ODNs and HIF-1α AS-ODNs 100 μg or 0 μg, X-ray irradiation 10 Gy or 0 Gy)

G	A (μg)	B (μg)	C (Gy)	Tumor weight^X^ (g)		AI (%)^X^		MVD^X^		Necrosis rate (%)^X^	
				Hep-2	Tu212	Hep-2	Tu212	Hep-2	Tu212	Hep-2	Tu212
G1	100	100	10	0.14 ± 0.02	0.25 ± 0.04	14.33 ± 0.67	27.24 ± 0.36	2.33 ± 1.33	4.00 ± 0.57	56.67 ± 3.33	46.67 ± 6.66
G2	100	100	0	0.47± 0.02	0.21 ± 0.05	18.99 ± 1.13	17.19 ± 1.16	9.33 ± 0.33	10.00 ± 0.57	11.67 ± 3.33	36.67 ± 8.81
G3	0	100	10	0.42 ±0.12	0.30 ± 0.06	4.32 ± 0.03	6.42 ± 0.18	7.00 ± 1.00	7.66 ± 0.33	5.00 ± 0.00	40.00 ± 15.28
G4	100	0	10	0.26 ± 0.04	0.26 ± 0.05	4.82 ± 0.06	11.09 ± 0.30	16.67 ± 2.66	13.33 ± 0.88	10.00 ± 0.00	43.33 ± 8.18
G5	0	0	10	0.24 ± 0.04	0.33 ± 0.06	2.84 ± 0.15	3.88 ± 0.57	10.33 ± 1.20	14.00 ± 0.57	5.00 ± 0.00	23.33 ± 8.33
G6	0	100	0	0.53 ± 0.22	0.30 ±0.01	4.03 ± 0.18	5.78 ± 0.98	15.33 ± 0.88	16.00 ± 0.57	11.67 ± 3.33	16.67 ± 3.33
G7	100	0	0	0.60 ± 0.20	0.18 ± 0.04	8.24 ± 0.35	9.03 ± 0.49	25.33 ± 2.40	27.67 ± 1.45	5.00 ± 0.00	11.67 ± 1.66
G8	0	0	0	0.59 ± 0.02	0.42 ± 0.06	1.78 ± 0.21	2.73 ± 0.71	41.33 ± 1.45	45.00 ± 2.88	5.00 ± 0.00	5.00 ± 0.00
a (*p*-value)				0.952	0.006	< 0.001	< 0.001	< 0.001	< 0.001	< 0.001	1.000
b (*p*-value)				0.592	0.379	< 0.001	< 0.001	< 0.001	< 0.001	< 0.001	0.043
c (*p*-value)				0.022	0.837	< 0.001	< 0.001	< 0.001	< 0.001	< 0.001	1.000
ab (*p*-value)				0.051	0.292	< 0.001	< 0.001	< 0.001	0.032	< 0.001	0.018
ac (*p*-value)				0.134	0.140	< 0.001	< 0.001	< 0.001	< 0.001	< 0.001	0.205
bc (*p*-value)				0.873	0.767	0.173	< 0.001	< 0.001	< 0.001	< 0.001	0.162
abc (*p*-value)				0.826	0.356	0.747	< 0.001	< 0.001	< 0.001	< 0.001	0.041

G: group, A: GLUT-1 AS-ODNs, B: HIF-1α AS-ODNs, C: X-ray irradiation.

a: main effect of GLUT-1 AS-ONDs, b: main effect of HIF-1α AS-ODNs, c: main effect of X-ray irradiation, ab: interaction effect between GLUT-1 AS-ONDs and HIF-1α AS-ODNs, ac: interaction effect between GLUT-1 AS-ONDs and X-ray irradiation, bc: interaction effect between HIF-1α AS-ODNs and X-ray irradiation, abc: interaction effect between GLUT-1 AS-ONDs and HIF-1α AS-ODNs and X-ray irradiation.

^X^ Mean ± Std(standard deviation).

### Effects of GLUT-1 AS-ODNs, HIF-1α AS-ODNs and 10Gy X-ray irradiation on xenografts GLUT-1 and HIF-1α mRNA levels

There was a synergistic interaction effect of the three treatments combined GLUT-1 AS-ODNs, HIF-1α AS-ODNs and 10Gy X-ray irradiation on decreasing the expression of GLUT-1 mRNA in Hep-2 and Tu212 xenografts 8 days and 12 days after treatment (*p* < 0.01). Only HIF-1α AS-ODNs decreased HIF-1α mRNA expression in Hep-2 xenografts significantly 8 days after treatment (*p* < 0.001). There were synergistic interaction effects of GLUT-1 AS-ODNs combined with HIF-1α AS-ODNs, GLUT-1 AS-ODNs combined with 10Gy X-ray irradiation, HIF-1α AS-ODNs combined with 10 Gy X-ray irradiation on decreasing the expression of HIF-1α mRNA in Hep-2 xenografts significantly 12 days after treatment (*P* = 0.021, *p* = 0.001, *p* < 0.001, respectively). In Tu212 xenografts, HIF-1α AS-ODNs and 10 Gy X-ray irradiation had synergistic interaction effects on decreasing the expression of HIF-1α mRNA 8 days and 12 days after treatment (*p* = 0.026,0.004, respectively) (Table 3) (Table 4).

**Table 3:** Observed GLUT-1 and HIF-1α expressions of xenografts 8 days after treatment in the 2^3^ factorial design with three independent parameters and their two levels (GLUT-1 AS-ODNs and HIF-1α AS-ODNs 100 μg or 0 μg, X-ray irradiation 10 Gy or 0 Gy)

G	A (μg)	B (μg)	C (Gy)	GLUT-1mRNA^X^		Glut-1 protein^X^		HIF-1α mRNA^X^		HIF-1α protein^X^	
				Hep-2	Tu212	Hep-2	Tu212	Hep-2	Tu212	Hep-2	Tu212
G1	100	100	10	0.15 ± 0.01	0.02 ± 0.01	0.32 ± 0.10	0.07 ± 0.03	0.36 ± 0.02	0.25 ± 0.03	0.36 ± 0.06	0.03 ± 0.00
G2	100	100	0	0.13 ± 0.01	0.03 ± 0.01	0.22± 0.11	0.07 ± 0.02	0.45 ± 0.05	0.40 ± 0.07	0.10 ± 0.05	0.06 ± 0.03
G3	0	100	10	0.71 ± 0.05	0.26 ± 0.03	0.85 ± 0.14	0.20 ± 0.09	0.45 ± 0.04	0.25 ± 0.05	0.60 ± 0.21	0.07 ± 0.03
G4	100	0	10	0.91 ± 0.08	0.12 ± 0.01	0.81 ± 0.20	0.09 ± 0.02	1.10 ± 0.09	0.59 ± 0.02	0.91 ± 0.08	0.11 ± 0.02
G5	0	0	10	1.09 ± 0.09	0.49 ± 0.07	0.67 ± 0.21	0.45 ± 0.03	1.18 ± 0.16	0.67 ± 0.05	0.63 ±0.12	0.22 ± 0.04
G6	0	100	0	0.34 ± 0.02	0.22 ± 0.05	0.14 ± 0.09	0.22 ±0.06	0.27 ± 0.01	0.34 ± 0.01	0.13 ± 0.02	0.10 ± 0.07
G7	100	0	0	0.20 ±0.02	0.11 ± 0.02	0.37 ± 0.06	0.11 ± 0.03	0.95 ± 0.06	0.92 ± 0.04	0.63 ± 0.14	0.17 ± 0.09
G8	0	0	0	1.00 ± 0.08	1.02 ± 0.15	1.07 ± 0.07	0.84 ± 0.09	1.01± 0.07	1.02 ± 0.14	0.86 ± 0.07	0.29 ± 0.06
a (*p*-value)				< 0.001	< 0.001	0.018	< 0.001	0.639	0.510	0.509	0.121
b (*p*-value)				< 0.001	< 0.001	0.002	< 0.001	< 0.001	< 0.001	< 0.001	0.003
c (*p*-value)				< 0.001	0.012	0.043	0.014	0.127	< 0.001	0.028	0.326
ab (*p*-value)				0.128	< 0.001	0.789	< 0.001	0.238	0.212	0.331	0.547
ac (*p*-value)				0.092	0.013	0.567	0.025	0.275	0.755	0.382	0.783
bc (*p*-value)				0.048	0.007	0.063	0.03	0.401	0.026	0.051	0.750
abc (*p*-value)				< 0.001	< 0.01	0.002	0.041	0.202	0.680	0.002	0.805

G: group, A: GLUT-1 AS-ODNs, B: HIF-1α AS-ODNs, C: X-ray irradiation.

a: main effect of GLUT-1 AS-ONDs, b: main effect of HIF-1α AS-ODNs, c: main effect of X-ray irradiation, ab: interaction effect between GLUT-1 AS-ONDs and HIF-1α AS-ODNs, ac: interaction effect between GLUT-1 AS-ONDs and X-ray irradiation, bc: interaction effect between HIF-1α AS-ODNs and X-ray irradiation, abc: interaction effect between GLUT-1 AS-ONDs and HIF-1α AS-ODNs and X-ray irradiation.

^X^ Mean ± Std(standard deviation).

**Table 4:** Observed GLUT-1 and HIF-1α expressions of xenografts 12 days after treatment in the 2^3^ factorial design with three independent parameters and their two levels (GLUT-1 AS-ODNs and HIF-1α AS-ODNs 100 μg or 0 μg, X-ray irradiation 10 Gy or 0 Gy)

The results detected 12 days after treatment of xenografts											
G	A (μg)	B (μg)	C (Gy)	GLUT-1mRNA^X^		Glut-1 protein^X^		HIF-1α mRNA^X^		HIF-1α protein^X^	
				Hep-2	Tu212	Hep-2	Tu212	Hep-2	Tu212	Hep-2	Tu212
G1	100	100	10	0.51 ± 0.02	0.20 ± 0.04	0.54 ± 0.08	0.04 ± 0.01	0.14± 0.01	0.26 ± 0.03	0.07 ± 0.02	0.03 ± 0.00
G2	100	100	0	0.62 ± 0.03	0.36 ± 0.02	0.82 ± 0.01	0.17 ± 0.02	0.31 ± 0.01	0.38 ± 0.01	0.19 ± 0.08	0.05 ± 0.01
G3	0	100	10	0.33 ± 0.02	0.59 ± 0.09	0.55 ± 0.15	0.26 ± 0.06	0.40 ± 0.03	0.34 ± 0.04	0.18 ± 0.06	0.09 ± 0.03
G4	100	0	10	0.50 ± 0.05	0.31 ± 0.04	0.76 ± 0.02	0.13 ± 0.02	0.34 ± 0.03	0.59 ± 0.08	0.32 ± 0.02	0.32 ± 0.13
G5	0	0	10	0.80 ± 0.06	0.44 ± 0.06	0.59 ± 0.18	0.57 ± 0.15	0.65 ± 0.08	0.54 ±0.01	0.42 ± 0.08	0.29 ± 0.07
G6	0	100	0	0.31 ± 0.03	0.50 ± 0.04	0.59 ±0.12	0.48 ± 0.08	0.21 ± 0.02	0.50 ± 0.02	0.28 ± 0.08	0.12 ± 0.00
G7	100	0	0	0.78 ± 0.05	0.48 ± 0.11	0.94 ± 0.10	0.39 ± 0.11	0.83 ± 0.04	1.01 ± 0.12	0.64± 0.15	0.29 ± 0.05
G8	0	0	0	1.00 ± 0.07	1.01 ± 0.11	1.10 ± 0.20	1.40 ± 0.39	1.00 ± 0.05	1.01 ± 0.11	0.66 ± 0.04	0.54 ± 0.15
a (*p*-value)				0.023	< 0.001	0.506	0.001	< 0.001	0.515	0.289	0.142
b (*p*-value)				< 0.001	0.01	0.036	0.004	< 0.001	< 0.001	< 0.001	0.001
c (*p*-value)				0.02	0.001	0.017	0.006	< 0.001	< 0.001	0.014	0.006
ab (*p*-value)				< 0.001	0.049	0.618	0.058	0.021	0.200	0.754	0.69
ac (*p*-value)				0.011	0.05	0.874	0.1570	0.001	0.623	0.727	0.217
bc (*p*-value)				0.001	0.006	0.386	0.1221	< 0.001	0.004	0.244	0.473
abc (*p*-value)				0.001	< 0.01	0.164	0.305	0.107	0.936	0.793	0.228

G: group, A: GLUT-1 AS-ODNs, B: HIF-1α AS-ODNs, C: X-ray irradiation.

a: main effect of GLUT-1 AS-ONDs, b: main effect of HIF-1α AS-ODNs, c: main effect of X-ray irradiation, ab: interaction effect between GLUT-1 AS-ONDs and HIF-1α AS-ODNs, ac: interaction effect between GLUT-1 AS-ONDs and X-ray irradiation, bc: interaction effect between HIF-1α AS-ODNs and X-ray irradiation, abc: interaction effect between GLUT-1 AS-ONDs and HIF-1α AS-ODNs and X-ray irradiation.

^X^ Mean ± Std(standard deviation).

### Effects of GLUT-1 AS-ODNs, HIF-1α AS-ODNs and 10Gy X-ray irradiation on xenografts GLUT-1 and HIF-1α protein levels

**Figure 2:**
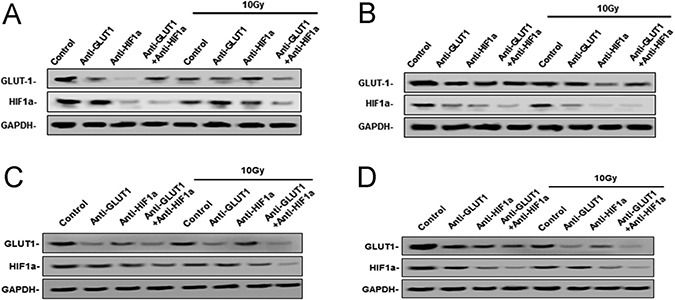
West-blotting analysis showed the protein levels of GLUT-1 and HIF-1α protein The expression of GLUT-1, HIF-1α protein in Hep-2 xenografts 8 days after treatment (**A**), 12 days after treatment (**B**) and Tu212 xenografts 8 days after treatment (**C**) and 12 days after treatment (**D**).

There was a synergistic interaction effect of the three treatments combined GLUT-1 AS-ODNs, HIF-1α AS-ODNs and 10Gy X-ray irradiation on decreasing the expression of GLUT-1 protein in Hep-2 and Tu212 xenografts 8 days after treatment (*p* = 0.002, *p* = 0.041, respectively). HIF-1α AS-ODNs and 10 Gy X-ray irradiation both decreased GLUT-1 protein expression in Hep-2 cells 12 days after treatment (*p* = 0.036, 0.017, respectively), surprisingly, GLUT-1 AS-ODNs alone didn't reduce the GLUT-1 protein levels. In Tu212 xenografts, GLUT-1 AS-ODNs, HIF-1α AS-ODNs or 10 Gy X-ray irradiation alone decreased GLUT-1 protein expression significantly (*p* = 0.001, 0.004, 0.006, respectively) 12 days after treatment, but there was no interaction effect. There was a synergistic interaction effect among the three treatments on decreasing HIF-1α protein levels in Hep-2 xenografts 8 days after treatment (*p* = 0.002). There was no equivalent synergistic interaction effect in Tu212 xenografts 8 days after treatment, or in Hep-2 or Tu212 xenografts 12 days after treatment. Only HIF-1α AS-ODNs alone significantly decreased HIF-1α protein levels in Tu212 cells 8 days after treatment (*p* = 0.003), HIF-1α AS-ODNs alone and 10Gy X-ray irradiation alone significantly decreased HIF-1α protein levels in Hep-2 xenografts (*p* < 0.001, 0.014, respectively) and Tu212 xenografts (*p* = 0.001, 0.006, respectively) 12 days after treatment. (Figure 2) (Table 3) (Table 4).

### Effects of GLUT-1 AS-ODNs, HIF-1α AS-ODNs and 10Gy X-ray irradiation on apoptosis, MVD, and necrosis in the xenografts

**Figure 3:**
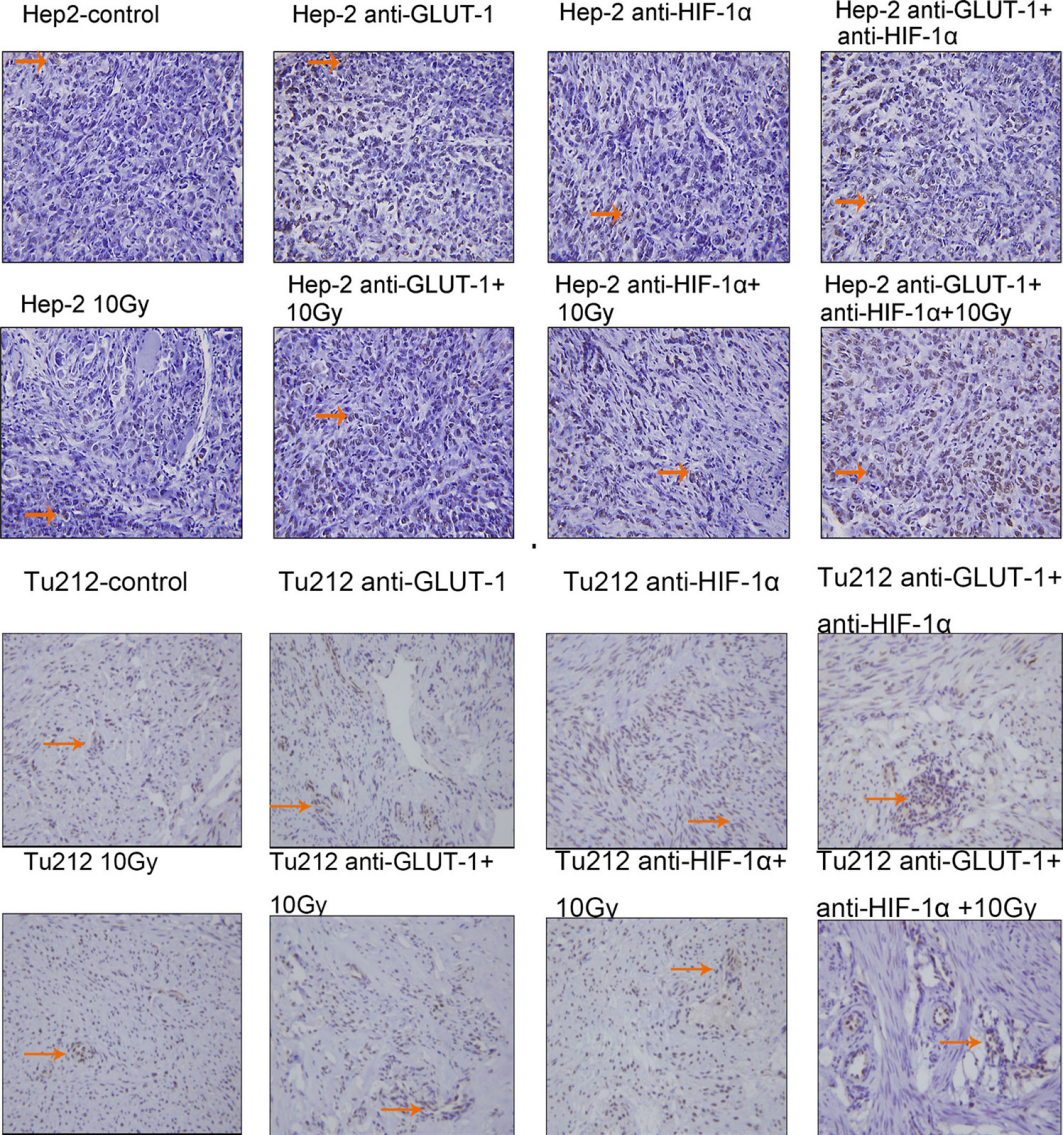
TUNEL positive staining showed that the nucleus was brown or brown-yellow, that is, apoptotic cells The arrows in the figure refer to apoptotic cells of Hep-2 and Tu212 xenografts 8 days after treatment. Apoptosis index were observed under an optical microscope (magnification, ×400).

**Figure 4:**
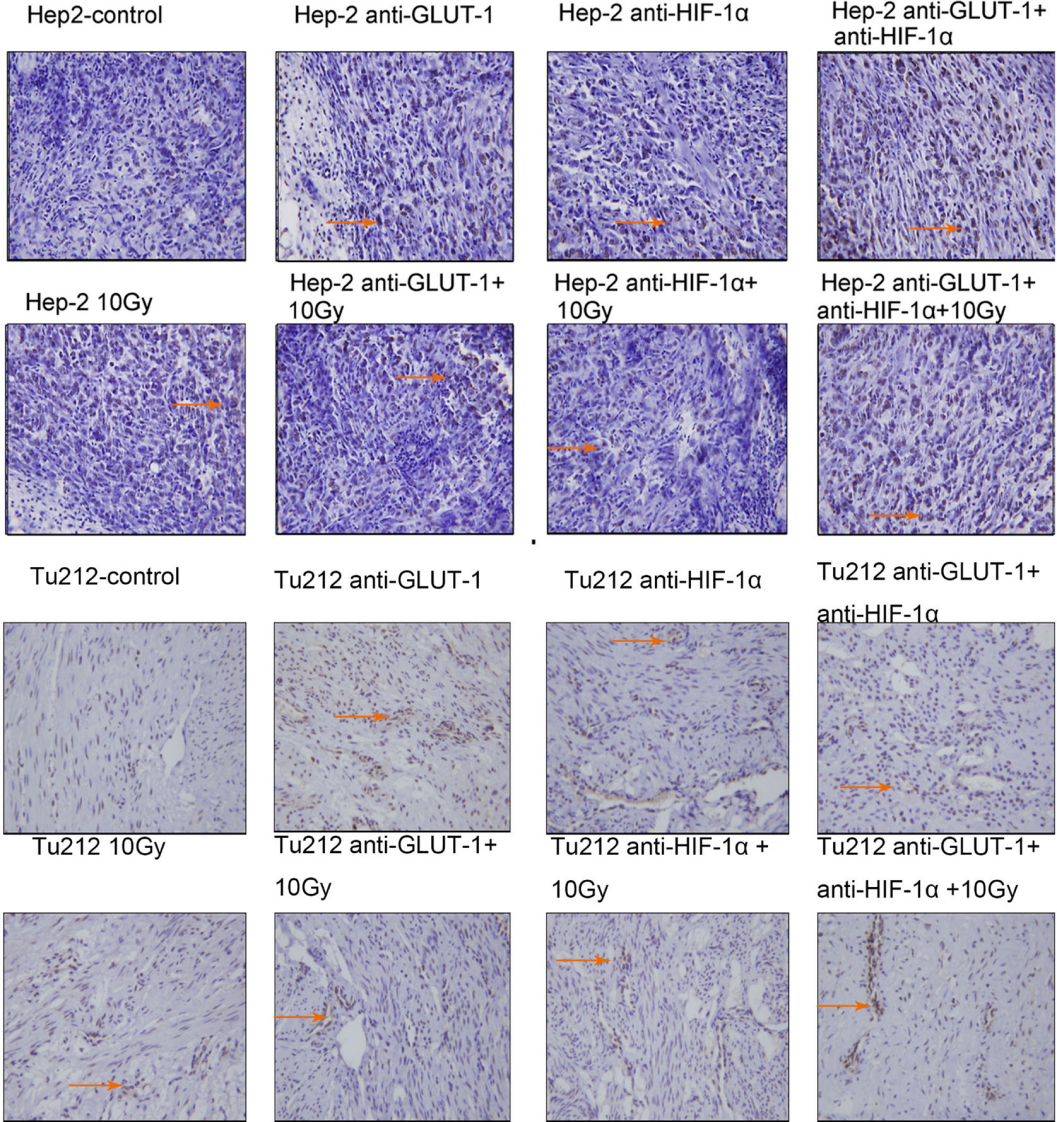
Apoptotic cells of Hep-2 and Tu212 xenografts 12 days after treatment pointed out with arrows Apoptosis index were observed under an optical microscope (magnification, ×400).

TUNEL positive staining showed that the nucleus was brown or brown-yellow, that is, apoptotic cells (Figure 3) (Figure 4). There was a synergistic interaction effect of the three treatments combined GLUT-1 AS-ODNs and HIF-1α AS-ODNs and 10Gy X-ray irradiation on increasing apoptosis in Hep-2 cells 8 days after treatment (*p* = 0.018). Although there was no synergistic interaction effect of the three treatments on apoptosis in Tu212 xenografts, GLUT-1 AS-ODNs combined with HIF-1α AS-ODNs, GLUT-1 AS-ODNs combined with 10Gy X-ray irradiation had synergistic interaction effects on increasing apoptosis in Tu212 xenografts 8 days after treatment (*P* < 0.001, *p* = 0.047, respectively) (Table 1). Interestingly, there was a synergistic interaction effect of the three treatments combined on increasing apoptosis in Tu212 cells 12 days after treatment (*p* < 0.001), there was no such effect on Hep-2 cells 12 days after treatment (*p* = 0.747). GLUT-1 AS-ODNs combined with HIF-1α AS-ODNs, GLUT-1 AS-ODNs combined with 10Gy X-ray irradiation had synergistic interaction effects on increasing apoptosis in Hep-2 xenografts 12 days after treatment (*P* < 0.001, *P* < 0.001, respectively) (Table 2).

**Figure 5:**
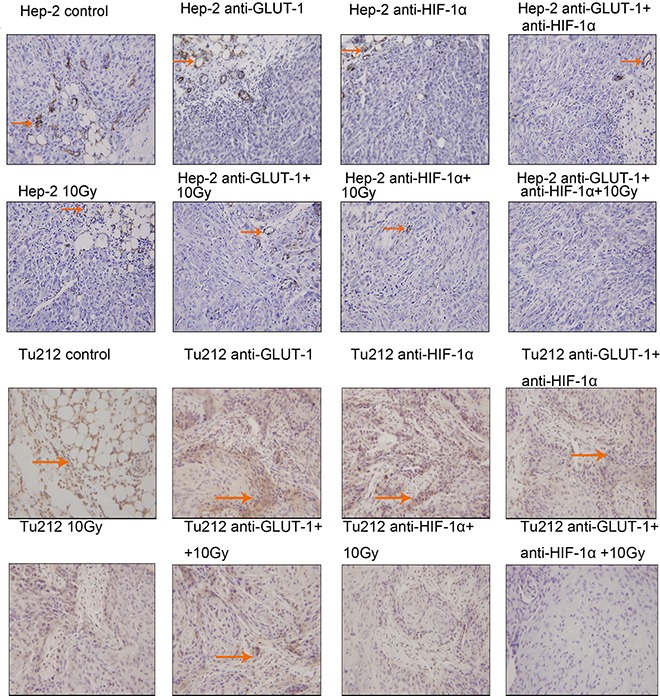
CD34 is the most sensitive marker of vascular endothelial cells, positive staining showed cytoplasm was light brown to brown The arrows in the figure refer to apoptotic cells of Hep-2 and Tu212 xenografts 8 days after treatment. Microvessel density were observed under an optical microscope (magnification, ×200).

**Figure 6:**
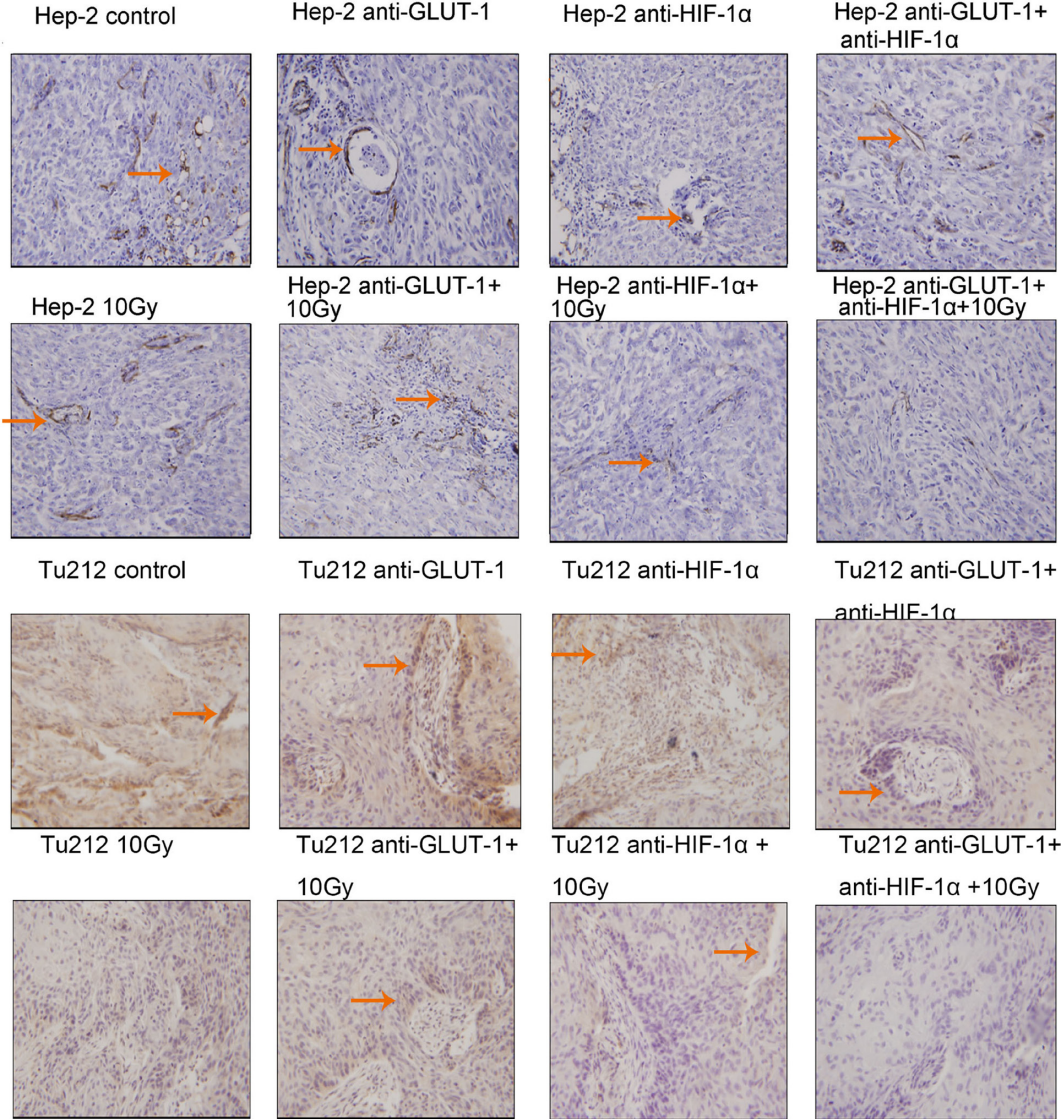
Microvessels of Hep-2 and Tu212 xenografts 12 days after treatment pointed out with arrows Microvessel density were observed under an optical microscope (magnification, ×200).

CD34 is the most sensitive marker of vascular endothelial cells, positive staining showed cytoplasm was light brown to brown (Figure 5) (Figure 6). There was a synergistic interaction effect of the three treatments combined GLUT-1 AS-ODNs and HIF-1α AS-ODNs and 10 Gy X-ray irradiation on decreasing MVD in Tu212 xenografts 8 days after treatment (*p* = 0.05), and in Hep-2 and Tu212 xenografts 12 days after treatment (*P* < 0.001, *P* < 0.001, respectively). GLUT-1 AS-ODNs combined with HIF-1α AS-ODNs, GLUT-1 AS-ODNs combined with 10 Gy X-ray irradiation, and HIF-1α AS-ODNs combined with 10 Gy X-ray irradiation had synergistic interaction effects on decreasing MVD in Hep-2 cells 8 days after treatment (*P* = 0.015, 0.002, 0.026, respectively) (Table 1) (Table 2).

**Figure 7:**
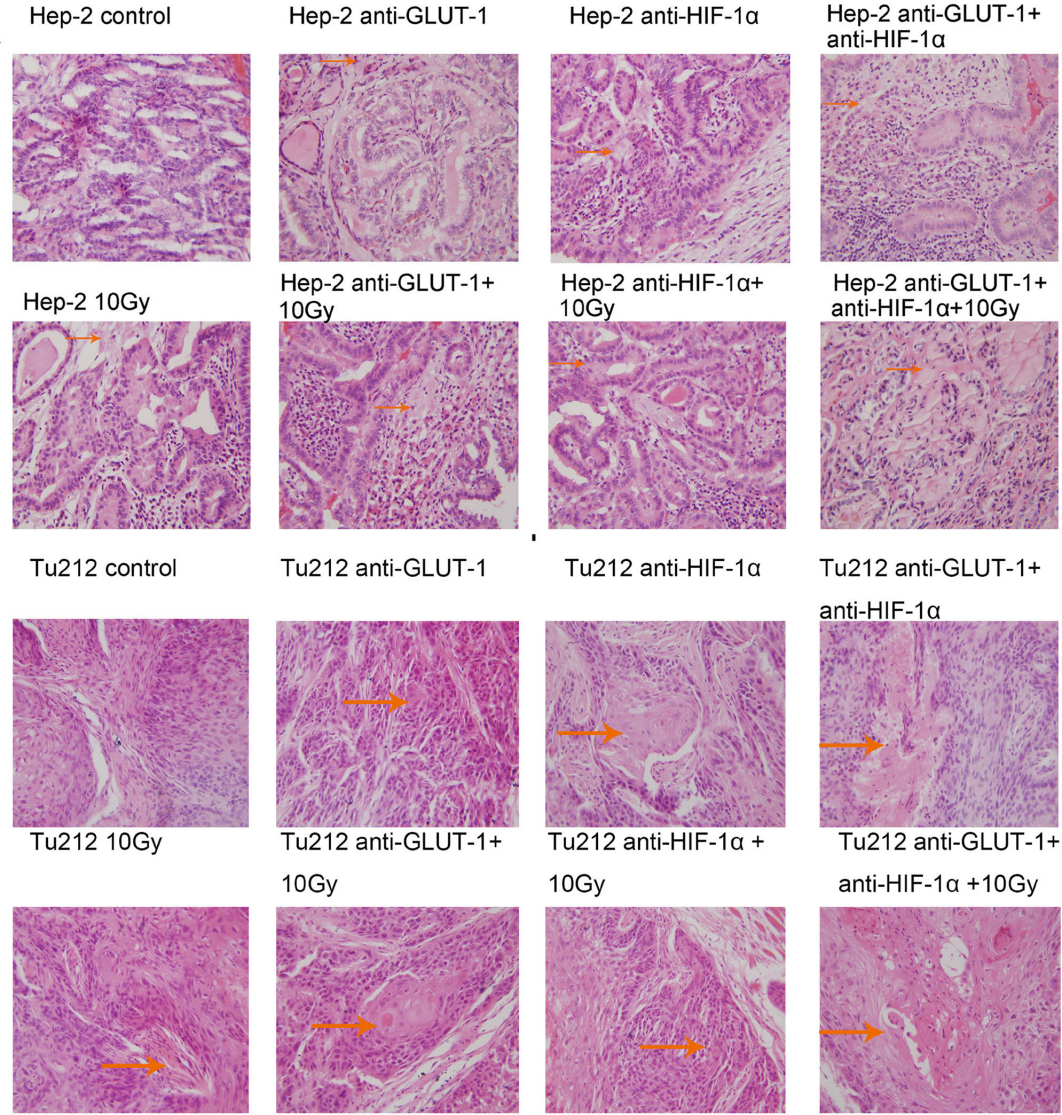
Cell membrane rupture, cell loss, nuclear concentration, nuclear fragmentation and nuclear dissolution were considered as tumor cells necrosis The arrows in the figure refer to necrosis of Hep-2 and Tu212 xenografts 8 days after treatment. Necrosis were observed under an optical microscope (magnification, ×400).

**Figure 8:**
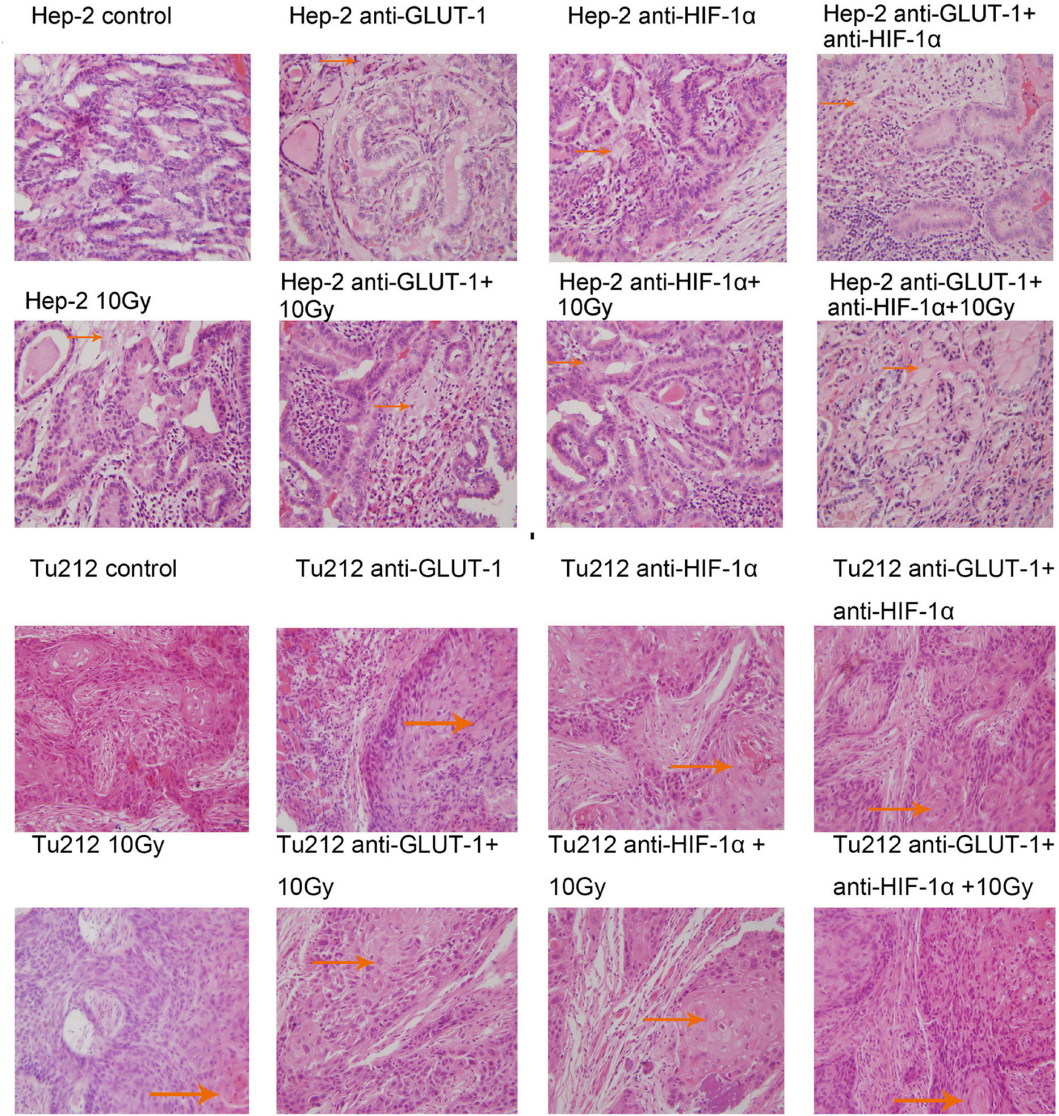
Necrosis of Hep-2 and Tu212 xenografts 12 days after treatment pointed out with arrows Necrosis were observed under an optical microscope (magnification, ×400).

Cell membrane rupture, cell loss, nuclear concentration, nuclear fragmentation and nuclear dissolution were considered as tumor cells necrosis (Figure 7) (Figure 8). There was a synergistic interaction effect of the three treatments combined on decreasing necrosis in Hep-2 and Tu212 xenografts 8 days after treatment (*P* < 0.001, *P* = 0.004, respectively), and in Hep-2 and Tu212 xenografts 12 days after treatment (*P* < 0.001, *P* = 0.041, respectively) (Table 1) (Table 2).

It indicated that the formulation, GLUT-1 AS-ODNs (100 μg) and HIF-1α AS-ODNs (100 μg) and 10Gy X-ray irradiation are optimized formulations with higher AI, higher necrosis rate and lower MVD of xenografts.

### Correlations among GLUT-1 expression, HIF-1α expression and SUVmax(T/N)

GLUT1 and HIF-1α mRNA expression revealed a significant correlation (Pearson's analysis) 8 days (*R* = 0.69, *p* = 0.001 in Hep-2 xenografts, *R* = 0.639, *p* = 0.001 in Tu212 xenografts)and 12 days after treatment (*R* = 0.672, *p* = 0.001 in Hep-2 xenografts, *R* = 0.599, *p* = 0.002 in Tu212 xenografts) . GLUT1 and HIF-1α protein levels revealed a significant correlation (Pearson's analysis) 8 days (*R* = 0.63, *p* = 0.001 in Hep-2 xenografts, *R* = 0.772, *p* = 0.001 in Tu212 xenografts) and 12 days after treatment (*R* = 0.691, *p* = 0.001 in Hep-2 xenografts, *R* = 0.73, *p* = 0.001 in Tu212 xenografts) .

The tumors grew in the right flanks of the mice, micro PET imaging of tumor xenografts demonstrated ^18^F-FDG uptake. However, GLUT1 and HIF-1α mRNA and protein expression showed no statistical correlations with SUVmaxT/N2 in both Hep-2 and Tu212 xenografts.

### Correlations between ^18^F-FDG accumulation and therapeutic effects

**Figure 9:**
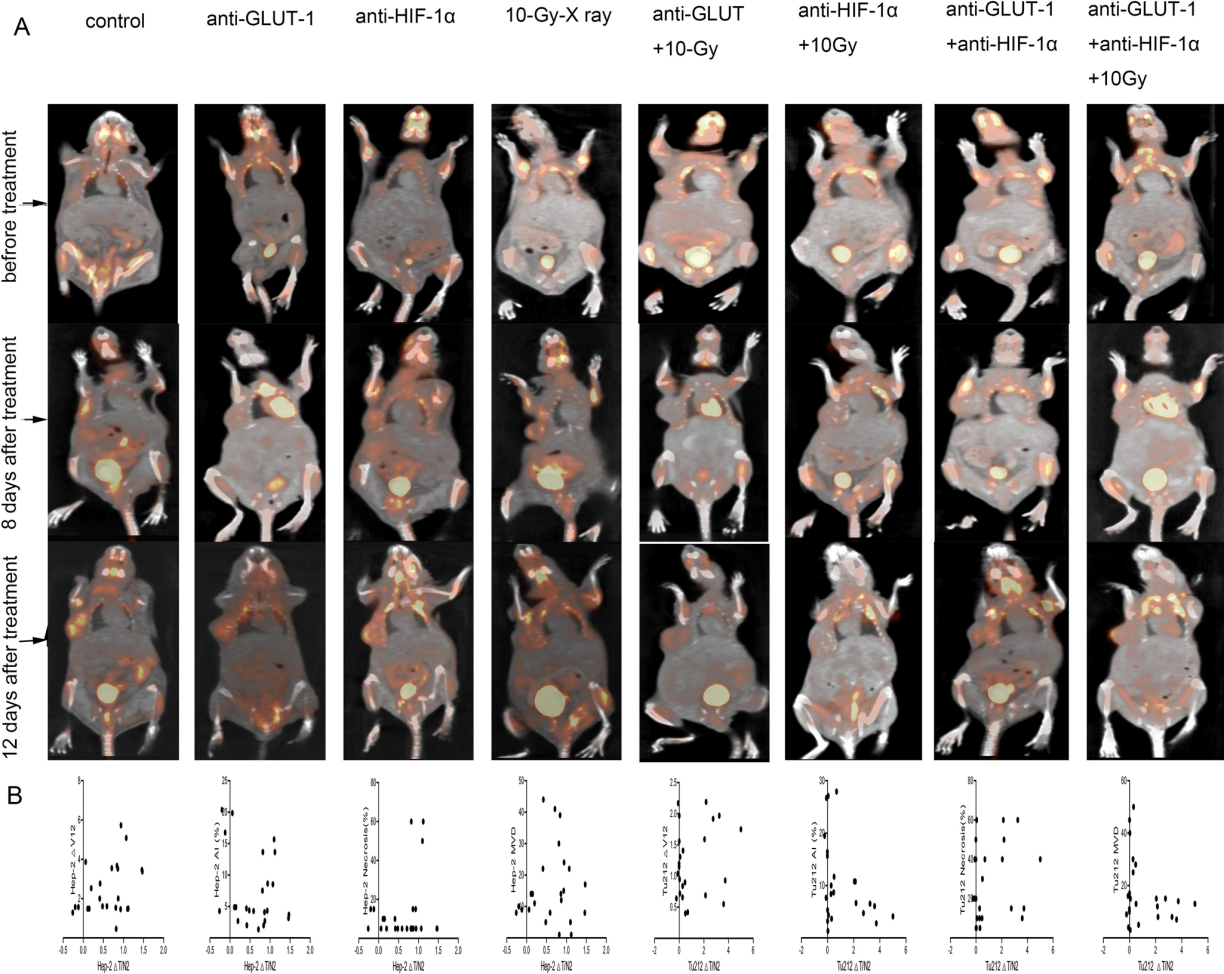
Tumors developed in the right flanks of mice (**A**). In both the Hep-2 and Tu212 groups (**B**), ∆SUVmaxT/N2 showed no statistical correlation with ∆V12, necrosis, apoptosis or MVD (all *p* > 0.05).

SUVmaxT/N was used to represent ^18^F-FDG accumulation. necrosis and apoptosis were considered to represent the therapeutic effect. We found that in both Hep-2 and Tu212 xenografts, SUVmaxT/N0 show no statistical significant difference between the groups. Neither SUVmaxT/N1, SUVmaxT/N2, ∆SUVmaxT/N1, ∆SUVmaxT/N2 nor ∆SUVmaxT/N12 show a statistical correlation with therapeutic effect (necrosis and apoptosis) in Hep-2 or Tu212 xenografts (Figure 9). Thus, we found no value in using SUVmaxT/N to evaluate therapeutic effects.

## DISCUSSION

Inhibiting the expression of HIF-1α to promote tumor radiosensitivity has been demonstrated in various preclinical studies [27, 28]. Tumor glucose metabolism can be targeted either directly by inhibiting enzymes and transporters involved in glucose metabolism or indirectly by anti-HIF-1 therapy [29]. In this study, we found GLUT-1 expression in the xenografts was inhibited significantly by HIF-1α AS-ODNs. HIF-1α AS-ODNs could decreased the expression of GLUT-1 mRNA and protein. It was consistent with other studies. In the study of Chen et al, they found inhibiting the expression of HIF-1α could decrease the expression level of GLUT-1, and thereby inhibited the volume and tumor weight of LOVO cell line xenografts [30]. Fan et al found that lentiviral vector-mediated RNA interference targeting HIF-1α significantly inhibited the expression of Glut-1 mRNA in Patu8988 pancreatic cancer cells [31]. In our study, GLUT-1 AS-ODNs alone didn't reduce the GLUT-1 protein levels in Hep-2 xenografts 12 days after treatment, however, in Tu212 xenografts 8 days and 12 days after treatment and in Hep-2 xenografts 8 days after treatment, GLUT-1 AS-ODNs alone decreased GLUT-1 protein expression significantly. This difference might be induced by changes in the stabilities of GLUT-1 mRNA.

Interfering with glucose metabolism in cancer cells to reduce levels of antioxidant metabolites could thus promote radiosensitivity [32]. Meijer et al found that targeting HIF-1 and glucose metabolism impacted the tumor microenvironment, and sensitized various solid tumors to irradiation [32].

Our data indicated a synergistic interaction effect among the three treatments on increasing apoptosis in Hep-2 and Tu212 xenografts. Some studies have shown that inhibiting the expression of HIF-1α or GLUT-1 alone increased tumor cell apoptosis [33–36]. Our results presented here showed that simultaneous inhibition of GLUT-1 and HIF-1α promoted tumor cell apoptosis more effectively than did inhibition of GLUT-1 or HIF-1α alone.

Angiogenesis could stimulate malignant tumor occurrence, development, invasion and metastasis. However, there have been no reports regarding the role of combined inhibition of HIF-1α and GLUT-1 on angiogenesis in laryngeal squamous carcinoma. In this study, there was a synergistic interaction effect of the three treatments on decreasing MVD in Hep-2 and Tu212 xenografts. Some studies have shown that downregulation of HIF-1α alone decreased intratumoral MVD [37, 38]. However, others have found that HIF-1α and GLUT-1 were not correlated with MVD in patients with locally advanced cervical cancer [39]. In the present study, the results showed that simultaneous inhibition of GLUT-1 and HIF-1α decreased MVD more effectively than did inhibition of GLUT-1 or HIF-1α alone in laryngeal cancer.

A synergistic interaction effect of the three treatments on increasing necrosis was also seen in Hep-2 and Tu212 xenografts. Some studies have demonstrated that inhibition of HIF-1 or GLUT-1 alone increased tumor cell necrosis [40, 41]. However, Petty et al found no significant correlation between GLUT-1 and the percentage of necrosis in canine osteosarcoma [42]. In the present study, the results showed that simultaneous inhibition of GLUT-1 and HIF-1α increased necrosis more effectively than did inhibition of GLUT-1 or HIF-1α alone in laryngeal cancer.

Although GLUT-1 AS-ODNs and HIF-1α AS-ODNs did not influence the weight or volume of the xenografts, they did increase tumor AI, increase tumor necrosis and decrease tumor MVD. GLUT-1 AS-ODNs and HIF-1α AS-ODNs might increase the radiosensitivity of Hep-2 and Tu212 xenografts.

In our experiments, GLUT1 and HIF-1α mRNA, and protein levels showed significant linear correlations, however, neither GLUT1 nor HIF-1α expression was correlated with SUVmaxT/N. Our results were similar to those of other studies in which ^18^F-FDG uptake did not correlate with GLUT-1 [43, 44], or HIF-1α expression [45]. However, other studies demonstrated that ^18^F-FDG uptake was correlated with HIF-1α [46], and GLUT-1 expression [45]. Yamada et al found that in early stage tumor, FDG uptake was associated with the expression of GLUT-1 and HIF-1, however, there were no correlations in the later-stage tumor [47]. These differences are likely explained by factors such as the accuracy of PET system quality control, image reconstruction algorithm and filtering, body composition, time interval between tracer injection and length of uptake period, plasma glucose level and partial volume effects [48, 49].

In the present study, neither SUVmaxT/N1, SUVmaxT/N2, ∆SUVmaxT/N1, ∆SUVmaxT/N2 nor ∆SUVmaxT/N12 showed a statistical correlation with therapeutic effects (necrosis, apoptosis). ^18^F-FDG PET for treatment monitoring was also influenced by the varying effectiveness of therapy in different stages of disease and in different tumor types [50]. There is controversy regarding the value of PET/CT in evaluating therapeutic effects on cancers. Some have stated that FDG PET/CT was a valuable tool for assessing treatment responses [21, 51]. ^18^F-FDG uptake was increased in hyperglycolyzed regions, but the exact mechanisms were complex and influenced by several microenvironmental parameters, such as the intracellular ^18^F-FDG phosphorylation capability, tumor oxygenation status, GLUT activity, blood flow and permeability [52–54]. Indeed, in most clinical experiments, FDG PET/CT was found to predict the treatment effect in tumors, however, this was not the case in preclinical experiments involving mouse tumor xenografts.

With respect to *in vivo* imaging, the proliferative activity of tumor cells has been shown to be more specific for cancers than has glucose metabolism. Thus, 3′-deoxy-3′-[^18^F] fluorothymidine (^18^F-FLT) may better reflect the proliferative capacity of tumor cells and may be useful in evaluating the therapeutic response [55], as it shows no uptake in inflamed tissues. Compared with ^18^F-FDG uptake in response to irradiation, ^18^F-FLT coincides more closely with the observed biological responses in cancer cell lines. Thus, in future study, we may use ^18^F-FLT as a PET tracer to investigate its value in predicting the treatment effect of laryngeal carcinoma.

The present study has several limitations, including the relatively low number of mice used per group, heterogeneity among the mice, the low frequency of drug administration and the influence of environmental factors (e.g air temperature) on PET/CT results. We accept that ^18^F-FDG uptake may also vary depending on tumor characteristics. SUV does not reflect the heterogeneity of a tumor, total lesion glycolysis (TLG) represents both the degree of ^18^F-FDG uptake and the size of the metabolically active tumor, and TLG has been proposed as a quantitative index of tumor metabolism. Hong et al demonstrated that TLG was a better predictor of survival than was SUV in patients with locally advanced esophageal cancer treated with radiotherapy [56]. Thus, we intend to perform subgroup analyses involving more mice and to evaluate the therapeutic effects on tumor xenografts using TLG in further investigations.

In conclusion, HIF-1α AS-ODNs and GLUT-1 AS-ODNs decreased HIF-1α and GLUT-1 levels, respectively, and HIF-1α AS-ODNs also decreased GLUT-1 level. Moreover, HIF-1α AS-ODNs and GLUT-1 AS-ODNs had synergistic interaction effects with 10 Gy X-ray irradiation on increasing tumor cells AI and necrosis, and decreasing MVD. These results provided evidence that HIF-1α and GLUT-1 may be promising targets for increasing tumor radiosensitivity, however, the interplay among radioresistance, HIF-1 and GLUT-1 expression warrants further investigation.GLUT1 and HIF-1α mRNA, and protein levels showed significant linear correlations, however, neither GLUT1 nor HIF-1α expression was correlated significantly with SUVmaxT/N. ^18^F-FDG SUVmaxT/N apparently had no value in assessing the therapeutic results of xenografts, because of the large variation in FDG tumor uptake. In the future, we will use more mice in our experiments and apply gene silencing methods potentially useful for developing new personalized therapeutic strategies for patients with laryngeal carcinoma. We will also use PET/CT with other radiotracer for further evaluation of the therapeutic effects.

## MATERIALS AND METHODS

### Ethics statement

Investigation has been conducted in accordance with the ethical standards and according to the Declaration of Helsinki and according to national and international guidelines and has been approved by the authors’ institutional review board.

### Reagents

The Hep-2 and Tu212 cell lines were purchased from the Cell Research Institute of Chinese Academy of Sciences (Shanghai, China) and XiangYa Central Experiment Laboratory (Hunan, China), respectively. Trypsin-EDTA solution and Lipofectamine 2000 were from Invitrogen Co. Ltd. USA. Sequences of the entire coding regions of GLUT-1 and HIF-1α were obtained from GenBank, and primers were designed using ClustalX and the Omega 2.0 software. CD34 was purchased from Proteintech (Chicago, IL, USA, Catalog No: 14486-1-AP). Four-week-old female nude mice (BALB/c AnNCrj-nu/nu) weighted 15–19 g were from Shanghai Sippr-BK Laboratory Animal Co. Ltd.

### Cell culture

Hep-2 and Tu212 cells were both cultured in RPMI-1640 (Gibco-BRL, Gaithersburg, MD, USA) supplemented with 10% heat-inactivated fetal bovine serum (Hyclone, Logan, UT, USA), 100 U/mL penicillin and 100 g/mL streptomycin at 37°C in a 5% CO_2_ atmosphere. Cells were trypsinised and harvested after reaching 80–90% confluence.

### Preparation of GLUT-1 AS-ODNs and HIF-1α AS-ODNs

After pcDNA3.1 plasmids (Xunjie biological technology Co. Ltd, Hangzhou, China) were digested with HindIII and XbaI, anti-GLUT-1 cDNA or anti-HIF-1α cDNA was subcloned into the vector pcDNA3.1 using T4 DNA ligase. The sequence of the GLUT-1 AS-ODN is 5′-ACAGAAAAGATGGCCACTGAG-3′ and HIF-1α AS-ODN is 5′-GCCGGCGCCCTCCAT-3′. The products were transfected into xenograft tumors using Lipofectamine 2000 reagent.

### Experimental design

The optimization of formulation variables was carried out using 2^3^ factorial design. Three independent formulation variables such as GLUT-1 AS-ONDs, HIF-1α AS-ODNs and X-ray irradiation each at two coded levels were designed., GLUT-1 AS-ODNs and HIF-1α AS-ODNs 100 μg or 0 μg, X-ray irradiation 10 Gy or 0 Gy. The effect of these factors was studied on tumor volume, tumor weight, MVD, AI, necrosis of the formulation as response variables.

### Nude mouse xenograft model

This experiment was performed in accordance with the institutional guidelines of the First Affiliated Hospital, College of Medicine, Zhejiang University. The animals were housed in a specific pathogen-free room under controlled temperature and humidity. Approximately 2 × 10^7^/mL × 0.2 mL of Hep-2 cells or Tu212 cells were inoculated subcutaneously into the right flank of mice. After 1 week, a grain-sized induration developed at the inoculation site, confirming that the xenograft model was established successfully. The mental state, food intake and activity of the mice were monitored daily. Micro PET/CT and X-ray radiation were performed under general anesthesia (7 ml/kg ip injection of 4% chloral hydrate).

The independent variables investigated were GLUT-1 AS-ODNs, HIF-1α AS-ODNs, and 10 Gy X-ray irradiation. The Hep-2 (*n* = 48) and Tu212 (*n* = 48) groups were treated as follows: (a) GLUT-1 AS-ODNs, HIF-1α AS-ODNs, and 10Gy X-ray irradiation combination group, the mice were injected peritumorally with 100 μg GLUT-1 AS-ODNs three times at 3-day intervals (on day 1, day 4, day 7), the tumors were exposed to 10 Gy X-ray once on day 5, and then 100 μg HIF-1α AS-ODNs was injected peritumorally into the tumors three times at 24 h intervals (on day5 immediately after irradiation, on day 6, day 7); (b) irradiation group, tumors were exposed to 10 Gy X-ray once on day 5; (c) GLUT-1 AS-ODNs group, the tumors were injected with GLUT-1 AS-ODNs on day1, day 4, day 7 as above; (d) HIF-1α AS-ODNs group, the tumors were injected with HIF-1α AS-ODNs on day 5, day 6, day 7 as above; (e) GLUT-1 AS-ODNs and irradiation combination group, the tumors were injected with GLUT-1 AS-ODNs and irradiated as above; (f) HIF-1α AS-ODNs and irradiation combination group, the tumors were injected with HIF-1α AS-ODNs and irradiated as above; (g) GLUT-1 AS-ODNs and HIF-1α AS-ODNs combination group, the tumors were injected with GLUT-1 AS-ODNs and HIF-1α AS-ODNs as above; (h) control group, the tumors were injected with RPMI-1640 on corresponding days. In each group, we randomly selected three mice to undergo microPET/CT three times (on day 0 before treatment, on day 8 and day 12 after treatment initiation). Mice that didn't undergo microPET/CT were sacrificed on day 8, others were sacrificed on day 12, these tumors were harvested and stored at −80°C .

### Xenograft volume

Tumor volumes were calculated every two days, based on caliper measurements of the short (a) and long (b) tumor diameters. Tumor volumes were calculated according to the formula V = 1/2 × a^2^ × b. The change in xenograft volume on day x was defined as ΔVx, ΔVx = (Vx–V0)/V0, where Vx is xenograft volume on day x, and V0 is the xenograft volume on day 0.

### Micro PET/CT

^18^F-FDG was synthesized by the PET Centre at our hospital. The animals were fasted overnight. ^18^F-FDG (250 μCi in 0.2 mL) was injected via the tail vein prior to PET scanning. The animals were anesthetized before PET/CT scanning. Then 1h later PET scanning was performed. Mice were placed prone in the center of Siemens Inveon combined microPET-CT scanner (Siemens Preclinical Solution USA, Inc., Knoxville, TN, USA) with limbs stretched. MicroCT scans were performed with an X-ray tube voltage of 80 kV, a current of 500 μA, an exposure time of 150 ms, and 120 rotation steps. A 10 min PET static acquisition was performed and the images were reconstructed using OSEM (ordered set expectation maximization) algorithm for 3D PET reconstruction. Images were analyzed with the Inveon Research Workplace 4.1 (Siemens, Erlangen, Germany). The standardized uptake value (SUV, the unit of SUV is g/ml) was determined by the formula SUV = [(RTA/cm3)/RID] × BW, where RTA is the measured radiotracer tissue activity (mCi), RID is the radiotracer injected dose (mCi), and BW is the mouse body weight (g). The maximum SUV (SUVmax) of the tumor (SUVmaxT) and opposite normal subcutaneous tissue (SUVmaxN) were recorded. SUVmaxT/N=SUVmaxT/SUVmaxN, SUVmax T/N on day 0, day8 and day 12 were referred as SUVmax T/N0, SUVmax T/N1, SUVmax T/N2, respectively. The change in ^18^F-FDG uptake for each tumor was determined using the following equations:

ΔSUVmaxT/N1=(SUVmaxT/N1-SUVmaxT/N0)/SUVmaxT/N0, ΔSUVmaxT/N2=(SUVmaxT/N2-SUVmaxT/N0)/SUVmaxT/N0, ΔSUVmax T/N12= (SUVmax T/N2- SUVmax T/N1)/ SUVmax T/N1.

### Reverse transcription polymerase chain reaction(RT-PCR)

Tumor tissue homogenates were collected and added Trizol according to the manufacturer's protocol, RNA was isolated and reverse transcribed. First,4.2 μg RNA, 2 μL Oligo(dT) (10 μM), 2 μL dNTP (2.5 mM) were mixed and ddH_2_O (RNase free) was added up to 14.5 μL. Reactions were incubated at 70°C for 5 min and kept on ice after centrifuging briefly, and then 5 × RT buffer (4 μl), HRP(RRI)/RNase inhibitor (0.5 μl), and M-MLV (1 μL) were added. After gentle mixing, the tubes were incubated at 42°C for 60 min, 95°C for 5 min. The PCR reagents included 10 μL 2× real-time PCR master mix (SYBR^®^-Green), 2 μL dNTP (2.5 mM), 0.25 μL Ex Taq, 2.5 μL 10 × Ex Taq E buffer, 1 μL cDNA, 9.25 μL ddH2O, in a total volume of 25 μL. The PCR primers used were as follows:

GAPDH sense, 5′-TGTTGCCATCAATGACCCCTT-3′, GAPDH antisense, 5′-CTCCACGACGTACTCAGCG-3′ (202 bp), GLUT-1 sense, 5′-GTCAACACGGCCTTCACTG-3′, GLUT-1 antisense, 5′-GGTCATGAGTATGGCACAACC-3′ (111 bp), HIF-1α sense, 5′-TTACAGCAGCCAGACGATCA-3′, HIF-1α antisense, 5′-CCCTGCAGTAGGTTTCTGCT-3′ (233 bp). To calculation differential gene expression, the 2−ΔΔCt formula was used.

### Western blotting

Tumor tissue homogenates were lysed in Radio Immunoprecipitation Assay (RIPA) lysis solution and were separated by gel electrophoresis and transferred to membranes. The membranes were blocked with 5% non-fat dry milk in TBST and then soaked in the primary antibody buffer, overnight at 4°C (GLUT-1 1:800 dilution(Proteintech, Chicago, IL, USA, cat no: 21829-1-AP), (HIF-1α 1:800 dilution (Proteintech, Chicago, IL, USA, cat no: 20960-1-AP). The membranes were soaked in secondary antibody buffer and incubated for 2 h at room temperature. The proteins were visualized using enhanced chemiluminescence and exposed to X-ray film. Protein expression was analyzed semi-quantitatively using the Gel Logic analysis system (Kodak, Rochester, NY, USA).

### Analysis of MVD

Sections were cut at 5 μm and placed on a Fisher Superfrost slide and dried for 1–2 h at room temperature. Slides were fixed with 4% polyoxymethylene and incubated with PBS containing 0.5% Triton X-100 successively to improve the penetration of the antibody. Primary antibody(CD34) was dropped on the coverslips and incubated overnight at 4°C according to the manufacturer's protocol. The slides were observed using a microscope, positive signals were pale brown or brown. To quantify the MVD, an area with maximum concentration of vessels was identified at low magnification (×100) and the three most intense fields were chosen for blood vessel counts at ×200 magnification. The mean of the three counts was calculated.

### Transferase-mediated dUTP digoxigenin nick end-labeling (TUNEL)

Tumor sections were assessed using the *In Situ* Cell Death Detection Kit-POD (Roche, Shanghai, China). Tissue sections were fixed using fixation solution and incubated with blocking solution. TUNEL reaction mixture was added according to the manufacturer's protocol. Staining was visualized under an optical microscope. Cells in which the nuclei were brown or brown-yellow were considered as positive. The total number of apoptotic cells at ×400 magnification in five randomly selected fields was counted. The AI was calculated as the percentage of positively stained cells, AI=number of apoptotic cells × 100/total number of nucleated cells.

### Haematoxylin and eosin (H&E)

Microscope slides with rehydrated tumor sections were prepared and dipped into a Coplin jar containing Mayer's hematoxylin for 30s, rinsed in H_2_O for 1 min, and stained with 1% eosin Y solution for 10–30s. Sections were dehydrated using two changes of 95% alcohol and two changes of 100% alcohol for 30s each. Each slice was assessed in three random microscopic fields (×400). The necrosis rate = necrosis area/total area in the field.

### Statistical analysis

Data were analyzed using SPSS software (ver 22.0). A 2^3^ factorial design was adopted in this study concerning with the effects of formulation variables and their interactions on response variables to obtain the optimized formulation. Pearson's analysis was used to evaluate the relationships among GLUT-1 and HIF-1α expression, ^18^F-FDG accumulation and therapeutic effects. *P* values < 0.05 were considered to indicate statistical significance.

### Ethical approval

All procedures performed in studies involving mice were in accordance with in accordance with institutional guidelines of the First Affiliated Hospital, College of Medicine, Zhejiang University and with appropriate institutional certification.

### Informed consent

Informed consent was obtained from all individual participants included in the study.

## Abbreviations

HIF-1α: hypoxia-inducible factor 1α
GLUT-1: glucose transporter-1
^18^F-FDG: 2′-deoxy-2′-[18F]fluoro-D-glucose
PET/CT: positron emission tomography-computed tomography
AS-ODNs: antisense oligodeoxynucleotides
MVD: microvessel density
AI: apoptosis index
SUV: standardized uptake value
RT-PCR: reverse transcription polymerase chain reaction
